# Why do we like what we like? When information flow matters

**DOI:** 10.3389/fnhum.2013.00731

**Published:** 2013-10-29

**Authors:** Luca F. Ticini, Diana Omigie

**Affiliations:** ^1^Wellcome Laboratory of Neurobiology, University College LondonLondon, UK; ^2^The Italian Society for Neuroaesthetics ‘Semir Zeki’Trieste, Italy; ^3^Laboratoire de Neurosciences Fonctionnelles et Pathologies (EA 4559), Université Lille-Nord de FranceLille, France; ^4^Hôpital de la Pitié-SalpêtrièreParis, France; ^5^Centre de Neuro-imagerie de RechercheParis, France; ^6^Centre de Recherche de l'Institut du Cerveau et de la Moëlle Épinière, UPMC-UMR 7225 CNRS - UMRS 975 INSERMParis, France

**Keywords:** aesthetics, emotion, music reward value, sensory cortex, connectivity

In a recent issue of *Science*, Salimpoor et al. (2013) reported a study in which they explored the neural correlates of aesthetic reward by measuring brain activity while people listened to a novel piece of music. Their results showed that the degree to which a song is found desirable is well predicted both by the level of activity in the nucleus accumbens and the degree of its functional connectivity with other areas, including the orbitofrontal cortex and the auditory cortices. Interestingly, in a previous study, Zeki and Stutters (2012) demonstrated that subjects' preference for kinetic stimuli correlates not just with the activity of the orbitofrontal cortex, but also with activity in a specific part of the visual cortex, namely area V5. Taken together, these two studies are important in highlighting the role of early sensory cortices in subjective preference, even if indirectly shown in the study by Salimpoor et al. (2013) where there was not a direct relationship between auditory activity and desirability. However, in terms of the synergistic relationship between early sensory cortices and reward regions like the nucleus accumbens, two possibilities remain. One is that greater local processing of stimuli with preferred configurations leads to greater connectivity with emotion areas, while another is that greater feedback from emotion areas to early sensory areas takes place during the processing of favored stimuli. In the absence of a definitive answer to this question in the literature, we propose that the latter option is the more plausible. Indeed, there is considerable evidence of feedback influences originating in distant emotion brain structures, such as the amygdala, on early sensory processing (Vuilleumier and Driver, 2007; Scharpf et al., 2010). Nonetheless, there is also support for the alternative view: indeed, low-level statistical regularities of biological significance may influence perceptual judgments and preference ratings (for a review see Graham and Redies, 2010). For instance, in the aesthetic domain, observers dislike images of abstract art that present unnatural statistics (Fernandez and Wilkins, 2008). We suggest that future investigations that consider the dynamics of information flow in response to aesthetic stimuli will provide insights into how their desirability arises. Such efforts will significantly contribute to characterizing the feedback and feedforward mechanisms involved in aesthetic judgments.

## References

[B1] Fernandez D. Wilkins A. J. (2008). Uncomfortable images in art and nature. Perception 37, 1098–1113 10.1068/p5814 18773732 PMC2581924

[B2] Graham D. J. Redies C. (2010). Statistical regularities in art: relations with visual coding and perception. Vision Res. 50, 1503–1150 10.1016/j.visres.2010.05.002 20580643

[B3] Salimpoor V. N. van den Bosch I. Kovacevic N. McIntosh A. R. Dagher A. Zatorre R. J. (2013). Interactions between the nucleus accumbens and auditory cortices predict music reward value. Science 340, 216–219 10.1126/science.1231059 23580531

[B4] Scharpf K. R. Wendt J. Lotze M. Hamm A. O. (2010). The brain's relevance detection network operates independently of stimulus modality. Behav. Brain Res. 210, 16–23 10.1016/j.bbr.2010.01.038 20122966

[B5] Vuilleumier P. Driver J. (2007). Modulation of visual processing by attention and emotion: windows on causal interactions between human brain regions. Phil. Trans. R. Soc. B Biol. Sci. 362, 837–855 10.1098/rstb.2007.2092 17395574 PMC2430001

[B6] Zeki S. Stutters J. (2012). A brain-derived metric for preferred kinetic stimuli. Open Biol. 2, 120001 10.1098/rsob.120001 22645660 PMC3352092

